# Association between SARS-CoV-2 infection and select symptoms and conditions 31 to 150 days after testing among children and adults

**DOI:** 10.1186/s12879-024-09076-8

**Published:** 2024-02-10

**Authors:** Yongkang Zhang, Alfonso Romieu-Hernandez, Tegan K. Boehmer, Eduardo Azziz-Baumgartner, Thomas W. Carton, Adi V. Gundlapalli, Julia Fearrington, Kshema Nagavedu, Katherine Dea, Erick Moyneur, Lindsay G. Cowell, Rainu Kaushal, Kenneth H. Mayer, Jon Puro, Sonja A. Rasmussen, Deepika Thacker, Mark G. Weiner, Sharon Saydah, Jason P. Block, PCORnet Network Partners, Faraz S. Ahmad, H. Timothy Bunnell, Olveen Carrasquillo, Elizabeth A. Chrischilles, Dimitri A. Christakis, Bernard P. Chang, Janis L. Curtis, Soledad A. Fernandez, Christopher B. Forrest, Daniel Fort, David A. Hanauer, Rachel Hess, Benjamin D. Horne, Philip Giordano, William Hogan, Abu Saleh Mohammad Mosa, James C. McClay, Samyuktha Nandhakumar, Bridget Nolan, Jihad S. Obeid, Brian Ostasiewski, Anuradha Paranjape, Lav Patel, Suchitra Rao, Patricia S. Robinson, William E. Trick, Jonathan C. Silverstein

**Affiliations:** 1 Department of Population Health Sciences, Weill Cornell Medical College, New York, NY USA; 2 Epidemic Intelligence Service, Centers for Disease Control and Prevention, Atlanta, GA USA; 3 CDC COVID-19 Response Team, Centers for Disease Control and Prevention, Atlanta, GA USA; 4 Louisiana Public Health Institute, New Orleans, LA USA; 5 Department of Population Medicine, Harvard Pilgrim Health Care Institute, Harvard Medical School, 401 Park Drive, Suite 401 East, Boston, MA USA; 6 Statlog, Montreal, Canada; 7 Peter O-Donnell Jr. School of Public Health, Department of Immunology, UT Southwestern Medical Center, Dallas, TX USA; 8 Fenway Institute, Fenway Health, Harvard Medical School, Boston, MA USA; 9 OCHIN, Inc., Portland, OR USA; 10 Department of Pediatrics, University of Florida College of Medicine, Gainesville, FL USA; 11 Nemours Cardiac Center, Nemours Children’s Health, Wilmington, Delaware USA

**Keywords:** SARS-CoV-2, Long-COVID, Electronic health record, COVID-19 pandemic

## Abstract

**Background:**

An increasing number of studies have described new and persistent symptoms and conditions as potential post-acute sequelae of SARS-CoV-2 infection (PASC). However, it remains unclear whether certain symptoms or conditions occur more frequently among persons with SARS-CoV-2 infection compared with those never infected with SARS-CoV-2. We compared the occurrence of specific COVID-associated symptoms and conditions as potential PASC 31- to 150-day following a SARS-CoV-2 test among adults and children with positive and negative test results.

**Methods:**

We conducted a retrospective cohort study using electronic health record (EHR) data from 43 PCORnet sites participating in a national COVID-19 surveillance program. This study included 3,091,580 adults (316,249 SARS-CoV-2 positive; 2,775,331 negative) and 675,643 children (62,131 positive; 613,512 negative) who had a SARS-CoV-2 laboratory test during March 1, 2020–May 31, 2021 documented in their EHR. We used logistic regression to calculate the odds of having a symptom and Cox models to calculate the risk of having a newly diagnosed condition associated with a SARS-CoV-2 positive test.

**Results:**

After adjustment for baseline covariates, hospitalized adults and children with a positive test had increased odds of being diagnosed with ≥ 1 symptom (adults: adjusted odds ratio[aOR], 1.17[95% CI, 1.11–1.23]; children: aOR, 1.18[95% CI, 1.08–1.28]) or shortness of breath (adults: aOR, 1.50[95% CI, 1.38–1.63]; children: aOR, 1.40[95% CI, 1.15–1.70]) 31–150 days following a SARS-CoV-2 test compared with hospitalized individuals with a negative test. Hospitalized adults with a positive test also had increased odds of being diagnosed with ≥ 3 symptoms or fatigue compared with those testing negative. The risks of being newly diagnosed with type 1 or type 2 diabetes (adjusted hazard ratio[aHR], 1.25[95% CI, 1.17–1.33]), hematologic disorders (aHR, 1.19[95% CI, 1.11–1.28]), or respiratory disease (aHR, 1.44[95% CI, 1.30–1.60]) were higher among hospitalized adults with a positive test compared with those with a negative test. Non-hospitalized adults with a positive test also had higher odds or increased risk of being diagnosed with certain symptoms or conditions.

**Conclusions:**

Patients with SARS-CoV-2 infection, especially those who were hospitalized, were at higher risk of being diagnosed with certain symptoms and conditions after acute infection.

**Supplementary Information:**

The online version contains supplementary material available at 10.1186/s12879-024-09076-8.

## Background

Studies have reported that 10–50% of individuals infected with SARS-CoV-2 develop new and persistent symptoms and conditions after the acute infection [1–4]. These new symptoms and conditions, sometimes referred to as post-acute sequelae of SARS-CoV-2 infection (PASC) or long-COVID, affect a wide range of organ systems [5]. Studies also have found that the occurrence of PASC is not uniform, with higher incidence among those who were older and had more severe SARS-CoV-2 infection (e.g., hospitalized) [6, 7].

Understanding the symptoms and conditions associated with SARS-CoV-2 infection has important clinical and public health implications. However, significant gaps remain. Although a few population-based studies have used large samples to examine PASC, they only focused on specific patient populations, such as US veterans and Medicare patients [1, 3, 6]. Prior studies of a generalizable population of adults have primarily included hospitalized COVID-19 patients without use of a control group, examined PASC related to a single organ system, focused on COVID-19 patients from a specific region, or did not adjust for some potential confounders between SARS-CoV-2 infection and PASC [8–11]. PASC among children and non-hospitalized adults has not been well characterized with large samples. Some studies used patient-reported data collected from surveys or interviews that can provide information about patient experience not routinely captured in healthcare data [12–14]. However, these patient-reported data may capture symptoms at only one point in time and may not account for symptoms and conditions before SARS-CoV-2 infection.

In this study, we used electronic health records (EHR) data from health systems participating in PCORnet, the National Patient-Centered Clinical Research Network [15], to examine whether select symptoms and conditions were associated with SARS-CoV-2 infection among adults and children compared with a control population of those who had only negative tests for SARS-CoV-2.

## Methods

### Study setting

PCORnet is a national research network of health systems that facilitates multi-site research using EHR data through use of a standardized common data model across all sites [16]. This study utilized data from 43 PCORnet sites participating in a national COVID-19 surveillance program funded by the Centers for Disease Control and Prevention (eTable 1). Starting in April 2020, sites have refreshed data at least monthly for a cohort of patients receiving care in their affiliated health systems who had a documented SARS-CoV-2 laboratory test, an International Classification of Diseases, Tenth Revision, Clinical Modification (ICD-10-CM) diagnostic code for a respiratory illness including but not limited to COVID-19, or a medication or procedure code for a COVID-19 vaccine or therapeutic.

### Study population

This study assessed all patients who had a SARS-CoV-2 laboratory test from March 1, 2020, through May 31, 2021. Patients were included in the study if they had encounter within a health system in the 540- to 31-day period prior to (baseline period) and in the 31- to 150-day period after (follow-up period) their index test date. This requirement facilitated identification of conditions and symptoms that were new after SARS-CoV-2 infection while accounting for baseline risk factors (e.g., age and baseline comorbidities) for PASC. Based on prior assessments of this population, we estimated that these restrictions led to the inclusion of 40% of all adults and 33% of all youth testing positive and 54% and 42% of those testing negative.

Patients were broadly stratified into two cohorts: a “youth cohort” including all children, adolescents, and young adults aged 0–19 (hereafter referred to as “youth cohort” or “children”) and an adult cohort (aged ≥ 20 years) based on their age at the index test date. Both age cohorts were further stratified based on hospitalization status associated with the SARS-CoV-2 laboratory test. Hospitalized patients included patients who were hospitalized on the day prior through the 16 days following the index test date.

### PASC symptoms and conditions

From prior studies, including another study using PCORnet data, we identified conditions and symptoms that may be more common among those testing positive for SARS-CoV-2 compared with those testing negative [1–4, 8, 17–19]. We examined this select set of conditions and symptoms in the 31- to 150-day period after the index SARS-CoV-2 test date between March 1, 2020, through May 31, 2021. Conditions identified using at least one ICD-10-CM diagnosis code included mental health conditions (e.g., anxiety, depression); chronic kidney disorders; diabetes mellitus type 1 or 2; hematologic disorders (e.g., venous thromboembolism); major cardiovascular events; neurological disorders (e.g., autonomic disorders); and respiratory diseases. We examined these conditions as potential PASC only in the adult cohorts as these conditions are extremely rare among patients aged less than 20 years. eTable 2 presents all the outcomes examined in this study.

Assessed symptoms included fatigue or muscle weakness, shortness of breath or dyspnea, cough, change in bowel habits, abdominal pain, headache, cognitive disorders, disorders of taste and smell, non-cardiac chest pain, heart rate abnormalities, sleep disorders and myalgias/arthralgias. From this list of symptoms, we created four symptom-related outcomes for both adult and youth cohorts, including 1) at least one symptom, which required only one ICD-10-CM code for any of the symptoms above; 2) three or more symptoms, which required at least 3 ICD-10-CM codes for the same or different symptoms; 3) fatigue or muscle weakness; and 4) shortness of breath or dyspnea. The outcome of three or more symptoms did not differentiate between symptoms codes on the same or different days – all were counted as part of this outcome. We examined the two single symptom outcomes because our previous study found that they were among the most prevalent symptoms after SARS-CoV-2 infection [4].

### Exposures and covariates

The exposure of interest was a positive SARS-CoV-2 test, defined as “positive”, “presumptive positive”, or “detected” (“positive viral test”), versus a negative SARS-CoV-2 test, defined as “negative” or “not detected” (“negative viral test”), on a rapid antigen (1% of patients) or polymerase chain reaction (PCR) tests (99% of patients). If patients had any positive SARS-CoV-2 viral test during the study period, they were analyzed as having only a positive test regardless of whether they had prior or subsequent negative tests. Patients categorized as having a negative viral test only had negative viral tests throughout the study period. The first positive or negative test date was defined as the index test date.

We controlled for several confounders in our regression analyses. For both children and adults, we controlled for age as a continuous variable, age squared to account for nonlinear effect of age, sex (female, male, and missing sex), race (Asian, Black, White, other race, missing), ethnicity (Hispanic, non-Hispanic, missing), weight class (children: BMI < 95th percentile, BMI ≥ 95th percentile, missing BMI; adults: BMI < 30 kg/m^2^, ≥ 30 kg/m^2^, missing BMI), and number of encounter in the health system in the 150- to 31-day period before the index date. For adults, we additionally controlled for combined comorbidity score [20] assessed based on conditions that occurred in the 540 to 7 days prior to the index date and current smoking status (current smoker; never, former or missing smoking), assessed based on the record closest to the index date in that same period. For hospitalized adults and children, we additionally controlled for length of stay, dexamethasone use, and mechanical ventilation during the hospitalization. Mechanical ventilation was identified from the index date through 16 days following the index date.

### Analyses

All analyses were conducted using distributed regression modeling, in which each site separately executed identical regression models. Based on the convergence of each regression at each site, results were either discarded or included in the meta-analysis. Once the convergence was assessed, results from the selected sites were combined using meta-analytic techniques (eTable 3). Across all models, convergence occurred in 32 to 42 sites in adult cohort analyses and 19 to 41 in youth cohort analyses; convergence occurred in fewer sites for models among patients who were hospitalized. The random-effects model based on the DerSimonian and Laird method was used to obtain pooled estimates [21].

Among adults, we examined each of the seven conditions in separate models. For each model, we excluded all patients who had a diagnostic code for the relevant condition that was the outcome for the model during the 540 to 31 days prior to the index date. We used Cox proportional hazard regression models, accounting for time from the beginning of the post-acute period (31 days post) to the earliest documentation of the first diagnostic code for each condition (event) and the end of the outcome period (150 days post-censoring). We controlled for all covariates described above in these models.

For the symptom outcomes, we did not exclude patients who had diagnostic codes for these symptoms during the baseline period as these symptoms are very common in routine clinical care. Instead, we controlled for the presence of these symptoms in the 150 to 31 days prior to index date. We used logistic regression models to assess the odds of having any of the four symptom outcomes associated with SARS-CoV-2 infection in the 31 to 150 days post index period. We controlled for the same covariates as we did in the condition outcome models, with the addition of a covariate indicating the presence of the relevant symptom or symptoms during the baseline period.

All analyses were done using the most recent version of SAS available at each of the sites executing analyses (Cary, NC). This activity was reviewed by CDC and conducted consistent with applicable federal law and CDC policy.

## Results

### Population characteristics

During March 1, 2020–May 31, 2021, we identified 3,091,580 unique adults aged 20 years or older meeting the inclusion criteria, including 316,249 with a positive viral test and 2,775,331 with only negative viral tests. We also identified 675,643 unique children 19 years or younger meeting the inclusion criteria, including 62,131 with a positive viral test and 613,512 with only negative tests (Table 1).

**Table 1:** Demographic and clinical characteristics of adults and children with a positive or negative SARS-CoV-2 test result

Characteristic	Adults (≥20 years old)				Children and young adults (0 -19 years)			
	Non-hospitalized		Hospitalized		Non-hospitalized		Hospitalized	
	Positive	Negative	Positive	Negative	Positive	Negative	Positive	Negative
No. with a SARS-CoV-2 test during March 1st, 2020—May 31st, 2021 AND had any diagnosis in the 540 to 31 days prior to the index date AND a diagnosis in the 31 to 150 days following the index date	270441	2102408	45808	672923	59374	520,816	2757	92696
**Age in years, mean (SD)**	48.9 (16.8)	52.8 (17.4)	59.9 (17.3)	55.9 (18.4)	10.2 (6.1)	8.3 (6.1)	10.3 (6.5)	8.9 (6.3)
**Age Group**, **years (N, % of patients**)								
0 - < 1	NA	NA	NA	NA	4969 (8.4)	51144 (9.8)	320 (11.6)	11649 (12.6)
1 - < 2	NA	NA	NA	NA	3769 (6.3)	50312 (9.7)	189 (6.9)	7415 (8.0)
2 - < 6	NA	NA	NA	NA	8797 (14.8)	114730 (22.0)	373 (13.5)	16778 (18.1)
6 - < 13	NA	NA	NA	NA	14983 (25.2)	142459 (27.4)	538 (19.5)	22058 (23.8)
13 - < 18	NA	NA	NA	NA	18512 (31.2)	115487 (22.2)	883 (32.0)	25433 (27.4)
18 - < 20	NA	NA	NA	NA	8344 (14.1)	46684 (9.0)	454 (16.5)	9363 (10.1)
20 - < 40	89892 (33.2)	571146 (27.2)	7361 (16.1)	172046 (25.6)	NA	NA	NA	NA
40 - < 55	75190 (27.8)	492470 (23.4)	8365 (18.3)	117210 (17.4)	NA	NA	NA	NA
55 - < 65	51300 (19.0)	421607 (20.1)	9867 (21.5)	128344 (19.1)	NA	NA	NA	NA
65 - < 75	34331 (12.7)	379716 (18.1)	10064 (22.0)	138562 (20.6)	NA	NA	NA	NA
75 - < 85	15191 (5.6)	185106 (8.8)	7176 (15.7)	85028 (12.6)	NA	NA	NA	NA
85 +	4537 (1.7)	52363 (2.5)	2975 (6.5)	31733 (4.7)	NA	NA	NA	NA
**Sex (N, % of patients)**								
Female	170017 (62.9)	1292859 (61.5)	24975 (54.5)	405346 (60.2)	30502 (51.4)	255450 (49.0)	1406 (51.0)	46404 (50.1)
Male	100409 (37.1)	809350 (38.5)	20829 (45.5)	267541 (39.8)	28868 (48.6)	265341 (51.0)	1351 (49.0)	46286 (49.9)
Other/Missing^a^	13 (0.0)	199 (0.0)	1–5 (0.0)	26 (0.0)	1–5 (0.0)	25 (0.0)	0 (0.0)	6–10 (0.0)
**Race (N, % of patients)**								
Asian	7158 (2.6)	61996 (2.9)	1140 (2.5)	17951 (2.7)	1620 (2.7)	15509 (3.0)	70 (2.5)	3179 (3.4)
Black or African American	42899 (15.9)	329438 (15.7)	11906 (26.0)	121268 (18.0)	9874 (16.6)	79953 (15.4)	686 (24.9)	16630 (17.9)
White	186703 (69.0)	1474317 (70.1)	24631 (53.8)	461579 (68.6)	37308 (62.8)	338040 (64.9)	1268 (46.0)	56192 (60.6)
Other^b^	21417 (7.9)	150712 (7.2)	6161 (13.4)	52651 (7.8)	6768 (11.4)	55905 (10.7)	582 (21.1)	12626 (13.6)
Missing^c^	12254 (4.5)	85945 (4.1)	1962 (4.3)	19471 (2.9)	3788 (6.4)	31405 (6.0)	151 (5.5)	4056 (4.4)
**Ethnicity (N, % of patients)**								
Hispanic	44275 (16.4)	201931 (9.6)	8455 (18.5)	63904 (9.5)	13739 (23.1)	83228 (16.0)	781 (28.3)	14557 (15.7)
Non-Hispanic	206370 (76.3)	1683892 (80.1)	34761 (75.9)	569136 (84.6)	42440 (71.5)	401034 (77.0)	1898 (68.8)	74749 (80.6)
Other	1491 (0.6)	23112 (1.1)	50 (0.1)	643 (0.1)	173 (0.3)	2147 (0.4)	1–5 (0.0)	353 (0.4)
Missing^c^	18305 (6.8)	193473 (9.2)	2538 (5.5)	39237 (5.8)	3014 (5.1)	34407 (6.6)	76–80 (2.8)	3035 (3.3)
**BMI (N, % of patients)**								
Obesity^d^	97082 (35.9)	661683 (31.5)	18889 (41.2)	243020 (36.1)	9372 (15.8)	74928 (14.4)	582 (21.1)	15767 (17.0)
Missing BMI	72210 (26.7)	511513 (24.3)	9946 (21.7)	115278 (17.1)	16826 (28.3)	135253 (26.0)	546 (19.8)	16783 (18.1)
**Number of visits in the 31 to 150 days before the index event, mean (SD)**	5.4 (6.8)	5.5 (6.8)	7.6 (9.0)	8.0 (8.4)	3.7 (5.7)	4.0 (6.3)	10.8 (13.4)	7.7 (9.9)
**Dexamethasone use (N, % of patients)**	8822 (3.3)	188156 (8.9)	16112 (35.2)	143911 (21.4)	634 (1.1)	52284 (10.0)	511 (18.5)	27072 (29.2)
**Length of hospital stay, days, mean (SD)**	NA	NA	7.3 (139.8)	1.1 (277.7)	NA	NA	6.2 (12.7)	4.6 (41.9)
**Mechanical ventilation (N, % of patients)**	690 (0.3)	4484 (0.2)	2116 (4.6)	18520 (2.8)	57 (0.1)	2275 (0.4)	158 (5.7)	4305 (4.6)
**Current smoker** ^e^** (N, % of patients)**	15923 (5.9)	176664 (8.4)	2257 (4.9)	59378 (8.8)	NA	NA	NA	NA
**Combined comorbidity score,** ^f^** mean (SD)**	1.0 (2.1)	1.3 (2.3)	2.7 (3.3)	2.1 (2.9)	NA	NA	NA	NA

*Abbreviations*: *BMI* Body mass index (calculated as weight in kilograms divided by height in meters squared), *NA* Not applicable

^a^Other/missing sex includes no information, unknown, and other

^b^Other race includes native Hawaiian or other pacific islander, American Indian or Alaska Native, multiple race, and all other races

^c^Missing race and ethnicity includes refuse to answer, no information, unknown, and missing values

^d^For children, obesity was defined as BMI greater or equal to 95th percentile; for adults, obesity was defined as BMI ≥ 30 kg/m2

^e^Smoking status was ascertained based on information in the EHR data. Smoking status was assessed for adults only

^f^Combined comorbidity score was defined based on Gagne et al.’s combined comorbidity score. Combined comorbidity was assessed for adults only

Individuals testing positive were older than those testing negative in both age cohorts across most care settings, although non-hospitalized adults who tested positive were younger than those who tested negative (mean age: 49 vs 53 years, *P* < 0.001). Among both age cohorts, compared to those with a negative test, more patients with a positive test were Black (26% vs 18% among adults, *P* < 0.001, and 25% vs 18% among children, *P* < 0.001) among hospitalized patients and Hispanic (17% vs 10% among adults, *P* < 0.001 and 23% vs 16% among children, *P* < 0.001) in both care settings. Adults with a positive test were more likely to have obesity in both care settings (5% absolute difference in both care settings, *P* < 0.001). Hospitalized children with a positive test were more likely to have obesity than those with a negative test (21% vs 17%, *P* = 0.01). Hospitalized patients who tested positive experienced longer length of stay, were more likely to be on mechanical ventilation, and were likely to receive dexamethasone compared to those who tested negative.

### Prevalence of symptoms among children and adults

Hospitalized patients with a positive viral test had higher prevalence of all symptom outcomes than those with a negative viral test in both age cohorts 31–150 days after a SARS-CoV-2 test (Table 2). Over half (53%) of hospitalized adults with a positive viral test had at least one symptom compared to 44% among those with a negative viral test. Shortness of breath was more prevalent among hospitalized adults who tested positive compared with those who tested negative (17% and 10%, respectively). Similar patterns were observed among children (Table 2). Prevalence of symptoms 31–150 days after SARS-CoV-2 test were similar between non-hospitalized patients testing positive and those testing negative in both age groups (Table 2).

**Table 2:** Prevalence of symptoms and incidence of conditions in 31–150 days following SARS-CoV-2 testing among adults and children with positive and negative SARS-CoV-2 test results

	**No./total No. (%)**			
**Prevalent symptoms and incident conditions**	**Non-hospitalized**		**Hospitalized**	
	**Positive**	**Negative**	**Positive**	**Negative**
**Adults (≥20 years)**				
Symptoms outcomes				
At least one symptom	105819/262400 (40.3)	822370/2020829 (40.7)	23989/44926 (53.4)	291712/662295 (44.0)
Three or more symptoms	17946/262400 (6.8)	148606/2020829 (7.4)	6843/44926 (15.2)	73005/662295 (11.0)
Fatigue or muscle weakness	17188/262400 (6.6)	126156/2020829 (6.2)	5454/44926 (12.1)	58508/662295 (8.8)
Shortness of breath	17450/262400 (6.7)	133948/2020829 (6.6)	7562/44926 (16.8)	64494/662295 (9.7)
Conditions outcomes				
Mental health conditions	14180/188941 (7.5)	116175/1426799 (8.1)	3165/30079 (10.5)	47330/441280 (10.7)
Chronic kidney disorders	2762/252853 (1.1)	31724/1940517 (1.6)	1874/35031 (5.3)	23936/579705 (4.1)
Diabetes type 1 or type 2	4245/225157 (1.9)	36944/1778370 (2.1)	2016/29502 (6.8)	19753/533887 (3.7)
Hematologic disorders	2248/265308 (0.8)	18770/2054644 (0.9)	2082/43284 (4.8)	18122/646387 (2.8)
Major adverse cardiovascular events	7028/238918 (2.9)	71936/1783138 (4.0)	3980/30945 (12.9)	52227/504033 (10.4)
Neurological disorders	8478/236441 (3.6)	83539/1791423 (4.7)	3252/34755 (9.4)	44248/542090 (8.2)
Respiratory diseases	8298/229162 (3.6)	73078/1742318 (4.2)	4463/32125 (13.9)	37561/527227 (7.1)
**Children and young adults (0 -19 years)**				
Symptoms outcomes				
At least one symptom	15145/58196 (26.0)	134017/511383 (26.2)	1196/2725 (43.9)	32343/91933 (35.2)
Three or more symptoms	1159/58196 (2.0)	10534/511383 (2.1)	227/2725 (8.3)	4841/91933 (5.3)
Fatigue or muscle weakness	1338/58196 (2.3)	10324/511383 (2.0)	166/2725 (6.1)	3674/91933 (4.0)
Shortness of breath	1345/58196 (2.3)	11730 /511383 (2.3)	179/2725 (6.6)	3676 / 91933 (4.0)

At least one symptom refers to at least one ICD-10 code: fatigue or muscle weakness, shortness of breath, cough, change in bowel habits, abdominal pain, headache, cognitive disorders, disorders of taste and smell, non-cardiac chest pain, heart rate abnormalities, sleep disorders, and myalgia and arthralgia. Three or more symptoms refer to at least 3 ICD-10 diagnosis codes for one or more symptoms. Mental health conditions include anxiety, depression, other mood disorders, overdose, psychosis, substance misuse, and suicide ideation/attempts. Chronic kidney disorders include chronic kidney disease and nephrotic and nephritic syndromes. Hematologic disorders include other venous thromboembolism and pulmonary embolism. Major adverse cardiovascular events include arrythmias, heart failure, intracerebral hemorrhage, ischemic infarction, myocardial infarction, myocarditis, subarachnoid hemorrhage, transient ischemic attack or other stroke. Neurological disorders include ataxia, autonomic dysfunction, dementia, encephalitis, myoneural disorders, parkinsonism, peripheral nerve disorders, and seizures. Respiratory disease includes asthma, chronic bronchitis, chronic obstructive pulmonary disease, hypoxemia, interstitial lung disease, pulmonary edema, pulmonary hypertension, and chronic respiratory failure. Condition outcomes were assessed among adults only as these conditions are extremely rare among children. No. is the number of persons with the given symptom or condition diagnosed 31 to 150 days after SARS-CoV-2 testing. For condition outcomes, we excluded patients who also had a condition in 540–31 days prior to the index test date; total No. = number of persons meeting the inclusion criteria. Therefore, total denominators of condition outcomes vary for each row because of removal of persons with conditions in the baseline period

### Association between SARS-CoV-2 infection and prevalent symptoms 31 to 150 days after testing among hospitalized children and adults

**Fig. 1:**
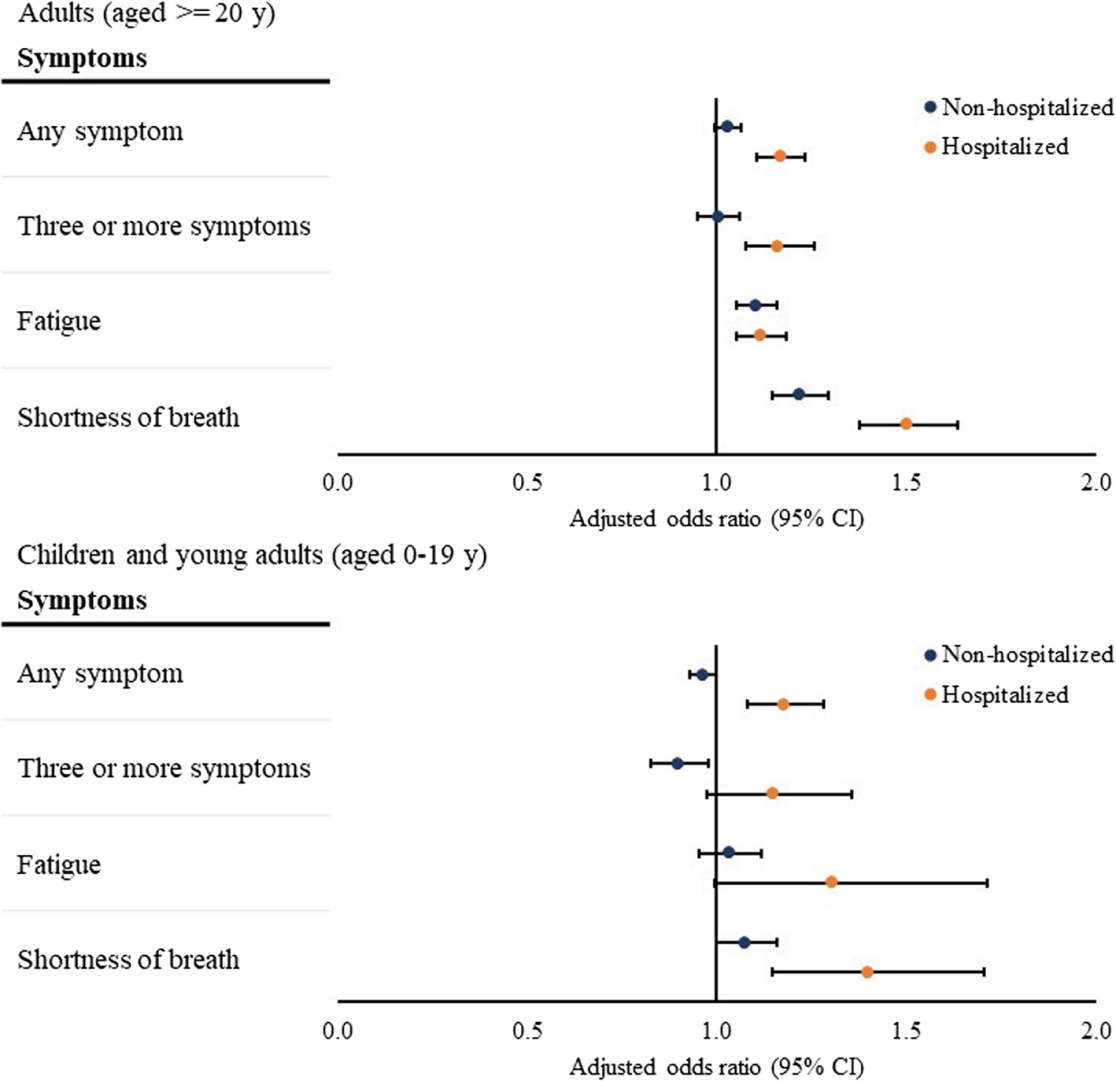
Association between SARS-CoV-2 infection and symptoms in 31 to 150 days after SARS-CoV-2 testing. Notes: Associations were assessed by comparing the presence of each outcome between patients with a positive viral test and those with a negative viral test, adjusting for baseline demographic and clinical characteristics as confounders using logistic regressions. The overall odds ratios were calculated using meta-analyses from site-specific estimates. At least one symptom refers to at least one symptom among fatigue or muscle weakness, shortness of breath, cough, change in bowel habits, abdominal pain, headache, cognitive disorders, disorders of taste and smell, non-cardiac chest pain, heart rate abnormalities, sleep disorders, and myalgia and arthralgia. Three or more symptoms refer to at least 3 different ICD-10 diagnosis codes for one or more symptoms. For both children and adults, regressions were adjusted for age, age squared, sex, race, ethnicity, weight class, number of encounters in the health systems. For adults, we additionally adjusted for combined comorbidity score based on the baseline health conditions and current smoking status. For hospitalized children and adults, we additionally controlled for length of stay, dexamethasone use, and mechanical ventilation during the hospitalization

Hospitalized adults with a positive test had increased odds of being diagnosed with at least one symptom (adjusted odds ratio [aOR], 1.17[95% CI, 1.11–1.23]), three or more symptoms (aOR, 1.16[95% CI, 1.08 – 1.26]), fatigue (aOR, 1.12[95% CI, 1.05 – 1.18]), or shortness of breath (aOR, 1.50[95% CI, 1.38–1.63]) 31 to 150 days after SARS-CoV-2 test (Fig. 1). Hospitalized children with a positive test had increased odds of being diagnosed with at least one symptom (aOR, 1.18[95% CI, 1.08–1.28]) or shortness of breath (aOR, 1.40[95% CI, 1.15–1.70]) 31–150 days after SARS-CoV-2 test (Fig. 1).

### Association between SARS-CoV-2 infection and prevalent symptoms 31 to 150 days after testing among non-hospitalized children and adults

Among non-hospitalized adults, those with a positive test had higher odds of being diagnosed with fatigue (aOR, 1.11[95% CI, 1.05–1.16]) or shortness of breath (aOR, 1.22[95% CI, 1.15–1.29]) 31–150 days after the index date compared with those with a negative test (Fig. 1). Among non-hospitalized children, those with a positive test had a decreased odds of being diagnosed with three or more symptoms (aOR, 0.90[95% CI. 0.83–0.98]) 31 to 150 days after the index date when compared with those with a negative test (Fig. 1).

### Incidence of new conditions among adults

Hospitalized adults with a positive test had higher incidence of type 1 or type 2 diabetes, hematologic disorders, major adverse cardiovascular events, and respiratory diseases, compared with those with a negative test (Table 2). The condition with the highest incidence among hospitalized adults with a positive test was respiratory diseases (14%), compared to 7% incidence among patients testing negative. Compared to non-hospitalized adults with a negative test, adults with a positive test had approximately similar incidence of type 1 or type 2 diabetes (2%), hematologic disorders (1%), mental health conditions (8%), and respiratory diseases (4%), and had lower incidence of the other conditions assessed (Table 2).

### Association between SARS-CoV-2 infection and new conditions among adults in 31 to 150 days after testing

**Fig. 2:**
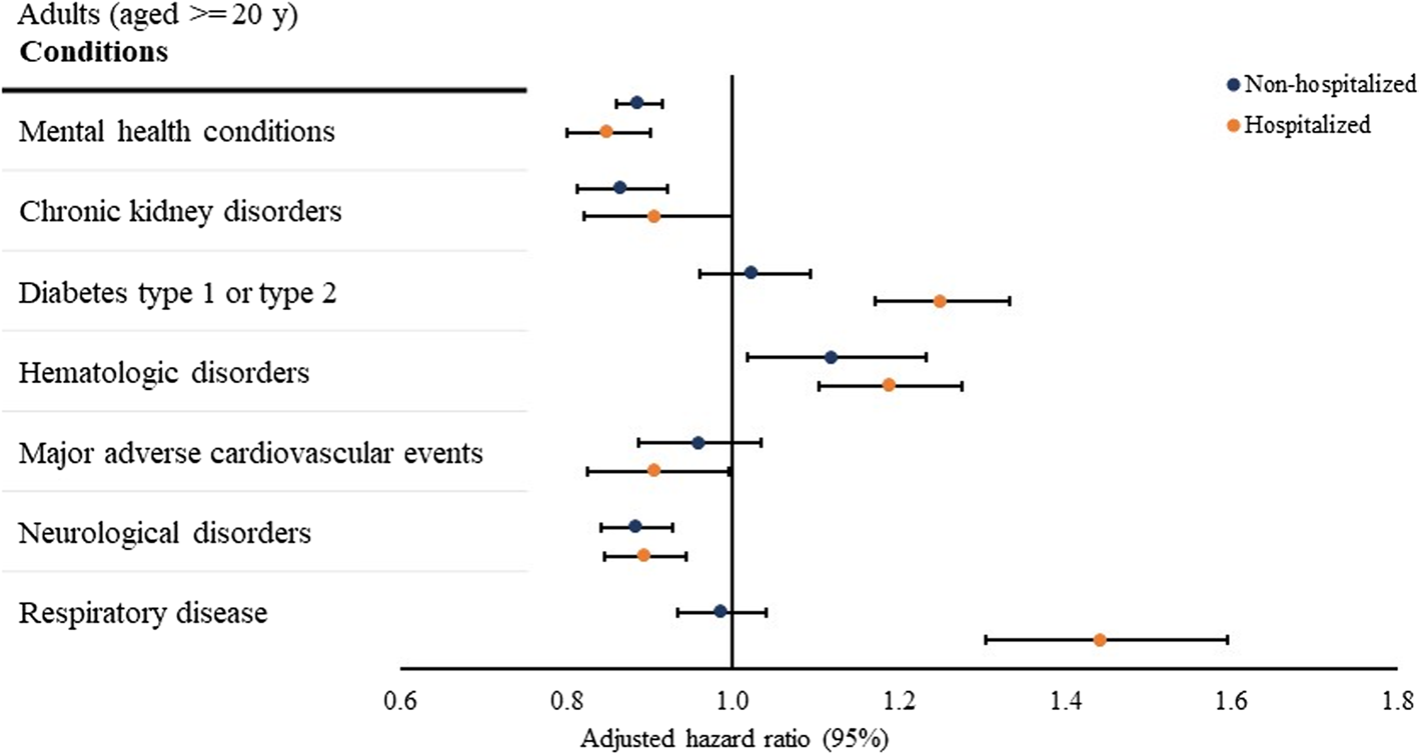
Association between SARS-CoV-2 infection and conditions in 31 to 150 days after SARS-CoV-2 testing. Notes: Associations were assessed using Cox proportional hazard regression models, accounting for time from the beginning of the post-acute period (31 days post) to the earliest presence of the first diagnostic code for each condition (event) and the end of the outcome period (150 days post-censoring). The overall hazard ratios were calculated using meta-analyses from site-specific estimates. Mental health conditions include anxiety, depression, other mood disorders, overdose, psychosis, substance misuse, and suicide ideation/attempts. Chronic kidney disorders include chronic kidney disease and nephrotic and nephritic syndromes. Hematologic disorders include other venous thromboembolism and pulmonary embolism. Major adverse cardiovascular events include arrythmias heart failure, intracerebral hemorrhage, ischemic infarction, myocardial infarction, myocarditis, subarachnoid hemorrhage, transient ischemic attack or other stroke. Neurological disorders include ataxia, autonomic dysfunction, dementia, encephalitis, myoneural disorders, parkinsonism, peripheral nerve disorders, and seizures. Respiratory disease includes asthma, chronic bronchitis, chronic obstructive pulmonary disease, hypoxemia, interstitial lung disease, pulmonary edema, pulmonary hypertension, and chronic respiratory failure. For both children and adults, regressions were adjusted for age, age squared, sex, race, ethnicity, weight class, number of encounters in the health systems. For adults, we additionally adjusted for combined comorbidity score based on the baseline health conditions and current smoking status. For hospitalized children and adults, we additionally controlled for length of stay, dexamethasone use, and mechanical ventilation during the hospitalization

The risk of being newly diagnosed with type 1 or type 2 diabetes (adjusted hazard ratio [aHR], 1.25[95% CI, 1.17–1.33]), hematologic disorders (aHR, 1.19[95% CI, 1.11–1.28]), or respiratory disease (aHR, 1.44[95% CI, 1.30–1.60]) were higher among hospitalized adults with a positive test compared with those with a negative test (Fig. 2), whereas the risk of being newly diagnosed with mental health conditions (aHR, 0.85[95% CI, 0.80–0.90]), major adverse cardiovascular events (aHR, 0.91[95% CI, 0.83–0.99]), or neurological disorders (aHR, 0.89[95% CI, 0.85–0.94]) was lower among hospitalized adults with a positive test relative to those with a negative test (Fig. 2).

Among non-hospitalized adults, those with a positive test had an increased risk of being newly diagnosed with hematologic disorders (aHR, 1.12[95% CI, 1.02–1.23]) and a decreased risk of being newly diagnosed with mental health conditions (aHR, 0.89[95% CI, 0.86–0.92]), chronic kidney disorders (aHR, 0.87[95% CI, 0.81–0.92]), or neurological disorders (aHR, 0.89[95% CI, 0.84–0.93]), compared with non-hospitalized adults with a negative test (Fig. 2).

## Discussion

Using EHR data of 3.7 million individuals who were tested for SARS-CoV-2 and received care from 43 PCORnet sites across the U.S., we identified that adults with a positive SARS-CoV-2 test were at increased odds of being diagnosed with certain symptoms and were at a higher risk of being newly diagnosed with certain conditions as potential PASC 31–150 days after testing, compared with patients who always tested negative for SARS-CoV-2. Hospitalized children with a positive SARS-CoV-2 test also were at increased odds of being diagnosed with symptoms, including shortness of breath, compared to those hospitalized children testing negative. Compared to previous studies, the major contributions of this study include (1) using a more generalizable population that included both adults and children; (2) conducting stratified analysis by hospitalization status; (3) controlling for a comprehensive set of baseline covariates as potential confounders between COVID-19 infection and PASC using longitudinal information in the EHR.

We found that differences in symptoms and conditions following SARS-CoV-2 positive and negative test results were more evident among hospitalized patients than non-hospitalized patients. These findings are consistent with literature reports showing that patients with more severe acute SARS-CoV-2 infection (i.e., hospitalized patients) have a higher risk of developing PASC conditions and symptoms [22]. We found relatively small differences in symptoms and conditions between non-hospitalized patients who tested positive and those who tested negative. For example, we found no symptom outcomes with higher odds among non-hospitalized children testing positive compared with those testing negative.

We did find some conditions that were more common among hospitalized adults testing negative, such as mental health conditions. While it is possible that these conditions are less common after SARS-CoV-2 infection, these differences also might reflect conditions for which patients testing negative were hospitalized. We could not define the primary reason for hospitalizations and thus could not control for the possibility that patients may have been hospitalized for conditions that persisted in the post-acute period of 31 to 150 days after index date. We did restrict these analyses to those patients who did not have these conditions during the baseline period.

Our results have important clinical and public health implications. Clinicians and public health agencies should monitor for the development and persistence of symptoms and conditions after COVID-19, especially among those who are hospitalized. The higher burden of PASC symptoms and conditions post-COVID, especially among those with severe disease, also should encourage investment in clinical and public health resources needed to deliver care to treat and prevent PASC, including ongoing support for trials underway to evaluate effectiveness of treatments for specific post-COVID conditions [23]. In addition, studies have indicated that COVID-19 vaccination may be protective against PASC, especially among patients who were fully vaccinated before infection [24–26]. Adherence to standard SARS-CoV-2 vaccination schedules may be an important practice to prevent PASC conditions and symptoms. Trials might be more impactful if they focused on patients initially hospitalized for COVID-19, because of the higher incidence among these patients.

This study has several limitations. First, using EHR data may have led to an underestimation of real prevalence and incidence of symptoms and conditions as we only observed diagnosis codes documented in health systems. Some patients in the control group may have tested positive at some point which was not captured in EHR (e.g., self-test at home). This would bias results toward the null. We were also unable to ascertain the exact length of baseline period for each patient; EHR data does not include information about when a patient begins or terminates their relationship with a health system. Therefore, patients may have different length of baseline periods, perhaps affecting our ability to ascertain prevalent conditions prior to a SARS-CoV-2 test. Similarly, using EHR data to ascertain PASC conditions and symptoms is dependent on patients’ encounters with the PCORnet affiliated health systems. If the patients testing positive vs. negative had a different probability of seeking healthcare in the 31- to 150-day period after their test, results may have been biased. However, especially for those hospitalized, we hypothesized that rates of follow-up should be similar; we also required that all patients have at least some follow-up in the 31- to150-day period after index, providing an opportunity for documentation of symptoms and conditions. This latter requirement may have led to a sicker population, considering that follow-up is more likely in patients who have more healthcare needs. Second, if patients testing positive vs. negative had a different probability of having baseline conditions documented in the EHR (up to 540 days prior), then ascertainment of incident conditions after testing may have differed. We again did not hypothesize that differences would be present, especially because the mean number of encounters prior to testing was similar (Table 1). Third, we defined symptoms or conditions as the occurrence of one ICD-10-CM diagnostic code 31 to 150 days following SARS-CoV-2 infection. This approach was used to enhance sensitivity but may have lower specificity. We also chose to aggregate some conditions together, such as type 1 and type 2 diabetes, because of some coding overlap in EHRs and because of limited power to detect differences of each individual condition separately, especially for type 1 diabetes. Of incidence cases of diabetes, over 90% were type 2 diabetes. Fourth, certain important covariates, such as vaccination status, were not included due to data limitations. Fifth, we used hospitalization within 16 days of a positive test for SARS-CoV-2 infection as a proxy for COVID-19 severity, which may have resulted in misclassification if patients with a positive test were hospitalized for reasons other than acute COVID-19 illness. If patients with asymptomatic infections (but hospitalized for other reasons) were more likely to have symptoms or conditions in the 31- to 150-day period than those with symptomatic infections, our findings of differences between patients testing positive vs. negative may be overestimated (biased away from the null). However, we hypothesized that patients with asymptomatic infections were likely more similar to those testing negative and thus results were likely biased toward the null. Sixth, hospitalized persons who tested negative for SARS-CoV-2 included those hospitalized for nonviral illness (e.g., pregnancy, trauma, and chronic conditions) and may have biased our estimates if these illnesses were associated with conditions or symptoms assessed in this study. Seventh, we broadly examined symptom outcomes in the population of children, adolescents, and young adults together; we considered this group together because we did not feel confident in the power of more narrow stratifications. Associations may differ between these age groups, and further work could clarify these outcomes. Finally, for covariates with missing values (e.g., sex and race), we adjusted for missing values as a separate category in the analyses. Imputing missing values may be a more robust approach.

## Conclusions

In conclusion, our findings suggest an association of post-acute sequelae of SARS-CoV-2 infection with higher severity of acute SARS-CoV-2 infection and highlight certain symptoms and conditions that are more common among patients testing positive for SARS-CoV-2. Future research is warranted to examine prevention and treatment of these symptoms and conditions to help patients recover from SARS-CoV-2 infection.

### Supplementary Information

**Additional file 1: eTable 1.** Institutions contributing data. **eTable 2.** Conditions and symptoms examined in the study. **eTable 3.** Number of Sites Included for Meta Analysis, by Outcome of the Regression Model.

## Data Availability

Data used in this project is not publicly available but can be requested from the PCORnet, the National Patient-Centered Clinical Research Network (https://pcornet.org/). All codes used for this query are available at GitHub at https://github.com/PCORnet-DRN-OC/Query-Details/tree/master/Long%20COVID%20Symptoms.

## Abbreviations

PASC: Post-acute sequelae of SARS-CoV-2 infection
EHR: Electronic health record
aOR: Adjusted odds ratio
aHR: Adjusted hazard ratio
PCR: Polymerase chain reaction
BMI: Body mass index
CDC: Centers for Disease Control and Prevention
