# Supplementary material for: Association between SARS-CoV-2 infection and select symptoms and conditions 31 to 150 days after testing among children and adults
Source: BMC Infect Dis. 2024 Feb 10;24:181. doi: 10.1186/s12879-024-09076-8 (PMC10859007; doi:10.1186/s12879-024-09076-8)
Supplement: Supplementary file 1 — Additional file 1: eTable 1. Institutions contributing data. eTable 2. Conditions and symptoms examined in the study. eTable 3. Number of Sites Included for Meta Analysis, by Outcome of the Regression Model. [file 12879_2024_9076_MOESM1_ESM.docx]

**Supporting information for**

Association between SARS-CoV-2 Infection and Select Symptoms and Conditions 31 to 150 Days After Testing among Children and Adults

[**eTable 1 Institutions contributing data** 2](#_Toc149302946)

[**eTable 2 Conditions and symptoms examined in the study** 4](#_Toc149302947)

[**eTable 3 Number of Sites Included for Meta Analysis, by Outcome of the Regression Model** 5](#_Toc149302948)

# **eTable 1 Institutions contributing data**

| **PCORnet sites** |
| --- |
| Duke University |
| Medical University of SC |
| University of North Carolina |
| Vanderbilt University Medical Center |
| Wake Forest Baptist Health |
| Allina Health |
| Intermountain Healthcare |
| Medical College of Wisconsin |
| University of Iowa Healthcare |
| University of Kansas |
| University of Missouri HC |
| University of Nebraska |
| University of Texas SW Medical Center |
| University of Utah |
| UMC New Orleans |
| Ochsner Health System |
| Children's Hospital Colorado |
| Children's Hospital of Philadelphia |
| Cincinnati Children's Hospital |
| Lurie Children's Hospital |
| Nationwide Children's Hospital |
| Nemours Children's Health |
| Seattle Children's Hospital |
| Columbia |
| Montefiore |
| Mount Sinai Health System |
| NYU Langone Medical Center |
| Weill Cornell Medicine |
| Cook County |
| Northwestern University |
| Fenway Health |
| Health Choice Network |
| OCHIN |
| Johns Hopkins University |
| Ohio State University |
| Penn State College of Medicine and Penn State Health Milton S. Hershey Medical Center |
| Temple University |
| University of Michigan |
| UPMC |
| AdventHealth |
| Orlando Health System |
| UF Health |
| University of Miami |

# **eTable 2 Conditions and symptoms examined in the study**

| **Symptoms examined** | **Conditions examined** |
| --- | --- |
| Fatigue or muscle weakness | Mental Health conditions |
| Shortness of breath or dyspnea | Chronic kidney disorders |
| Cough | Diabetes mellitus type 1 or type 2 |
| Change in bowel habits | Hematologic disorders |
| Abdominal pain | Major cardiovascular events |
| Headache | Neurologic disorders |
| Cognitive disorders | Respiratory diseases |
| Disorders of taste or smell |  |
| Non-cardiac chest pain |  |
| Heart rate abnormalities |  |
| Sleep disorders |  |
| Myalgias/arthralgias |  |

Note: The code list for the symptoms examined is posted at [https://github.com/PCORnet-DRN-OC/Query-Details/blob/master/Long%20COVID%20Symptoms/ CodeList_Long_COVID_Symptoms_Analytic_Query2_v1.0.xlsx](https://github.com/PCORnet-DRN-OC/Query-Details/blob/master/Long%20COVID%20Symptoms/%20CodeList_Long_COVID_Symptoms_Analytic_Query2_v1.0.xlsx). The code list for the conditions examined is posted at [https://github.com/PCORnet-DRN-OC/Query-Details/blob/master/Long%20COVID%20Descriptive/ CodeList_Long_COVID_Conditions_Analytic_Query2_v1.0.xlsx](https://github.com/PCORnet-DRN-OC/Query-Details/blob/master/Long%20COVID%20Descriptive/%20CodeList_Long_COVID_Conditions_Analytic_Query2_v1.0.xlsx).

# **eTable 3 Number of Sites Included for Meta Analysis, by Outcome of the Regression Model**

|  | Non-hospitalized | Hospitalized |
| --- | --- | --- |
| Adult (≥20 years) |  |  |
| Symptoms |  |  |
| At least one symptom | 42 | 38 |
| Three or more symptoms | 35 | 33 |
| Fatigue or muscle weakness | 39 | 33 |
| Shortness of breath | 36 | 33 |
| Conditions |  |  |
| Mental health conditions | 42 | 35 |
| Diabetes type 1 or type 2 | 38 | 33 |
| Hematologic disorders | 40 | 37 |
| Chronic kidney disorders | 36 | 32 |
| Major adverse cardiovascular events | 40 | 35 |
| Neurological disorders | 41 | 35 |
| Respiratory diseases | 40 | 35 |
| Children and young adults (0 -19 years) |  |  |
| Symptoms |  |  |
| At least one symptom | 40 | 36 |
| Three or more symptoms | 31 | 20 |
| Fatigue or muscle weakness | 34 | 19 |
| Shortness of breath | 36 | 21 |
